# Supplementary material for: Hot springs viruses at Yellowstone National Park have ancient origins and are adapted to thermophilic hosts
Source: Commun Biol. 2024 Apr 9;7:312. doi: 10.1038/s42003-024-05931-1 (PMC11003980; doi:10.1038/s42003-024-05931-1)
Supplement: Supplementary file 2 — Supplementary Fig. 1 [file 42003_2024_5931_MOESM2_ESM.pdf]

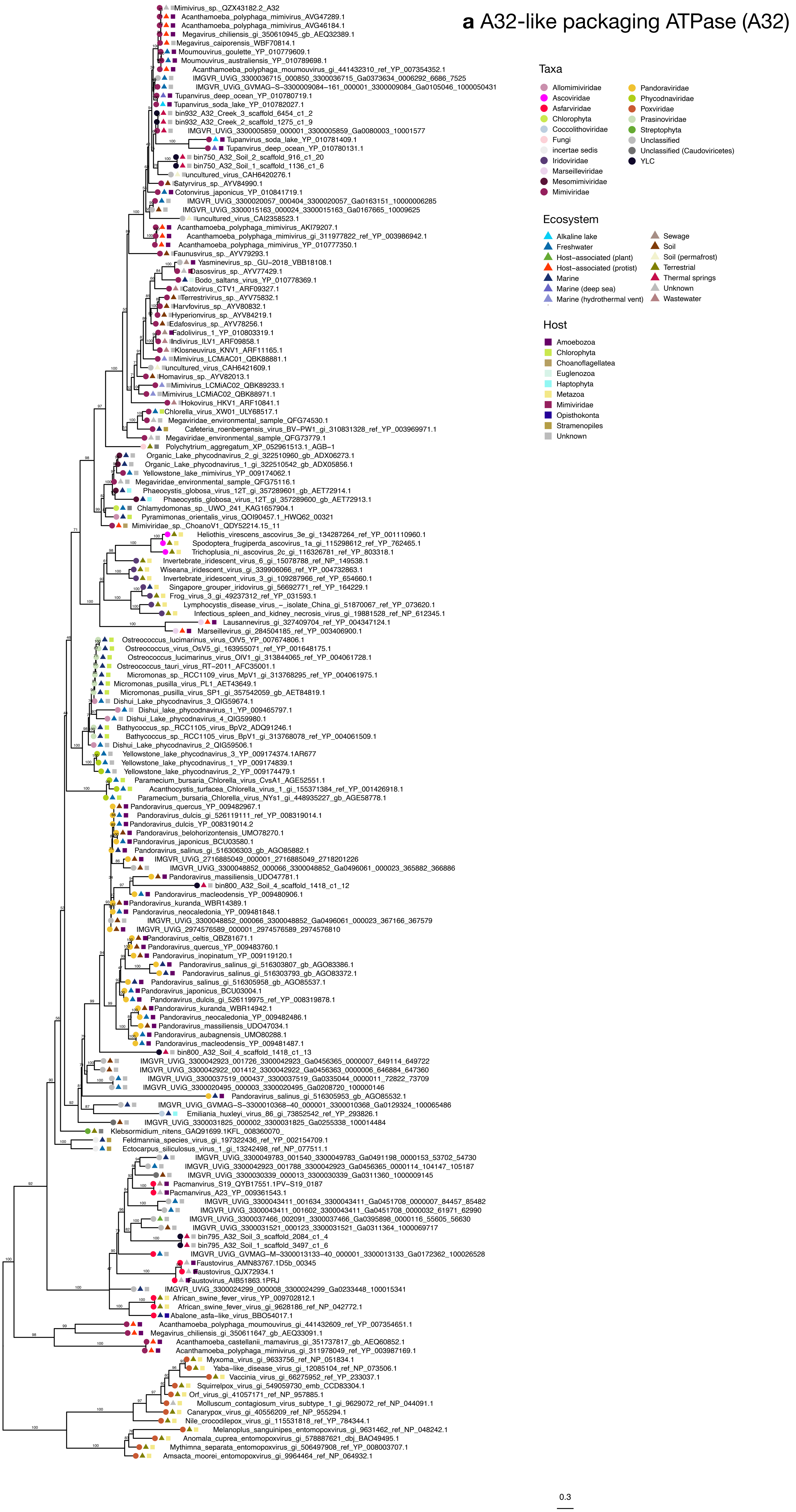
a A32-like packaging ATPase (A32)
Taxa
Allomimiviridae
Ascoviridae
Asfarviridae
Chlorophyta
Coccolithoviridae
Fungi
incertae sedis
Iridoviridae
Marseilleviridae
Mesomimiviridae
Mimiviridae
Pandoraviridae
Phycodnaviridae
Poxviridae
Prasinoviridae
Streptophyta
Unclassified
Unclassified (Caudoviricetes)
YLC
Ecosystem
Alkaline lake
Freshwater
Host–associated (plant)
Host–associated (protist)
Marine
Marine (deep sea)
Marine (hydrothermal vent)
Sewage
Soil
Soil (permafrost)
Terrestrial
Thermal springs
Unknown
Wastewater
Host
Amoebozoa
Chlorophyta
Choanoflagellatea
Euglenozoa
Haptophyta
Metazoa
Mimiviridae
Opisthokonta
Stramenopiles
Unknown
Mimivirus_sp._QZX43182.2_A32
Acanthamoeba_polyphaga_mimivirus_AVG47289.1
Acanthamoeba_polyphaga_mimivirus_AVG46184.1
Megavirus_chiliensis_gi_350610945_gb_AEQ32389.1
Megavirus_caiporensis_WBF70814.1
Moumouvirus_goulette_YP_010779609.1
Moumouvirus_australiensis_YP_010789698.1
Acanthamoeba_polyphaga_moumouvirus_gi_441432310_ref_YP_007354352.1
IMGVR_UViG_3300036715_000850_3300036715_Ga0373634_0006292_6686_7525
IMGVR_UViG_GVMAG–S–3300009084–161_000001_3300009084_Ga0105046_1000050431
Tupanvirus_deep_ocean_YP_010780719.1
Tupanvirus_soda_lake_YP_010782027.1
bin932_A32_Creek_3_scaffold_6454_c1_2
bin932_A32_Creek_2_scaffold_1275_c1_9
IMGVR_UViG_3300005859_000001_3300005859_Ga0080003_10001577
Tupanvirus_soda_lake_YP_010781409.1
Tupanvirus_deep_ocean_YP_010780131.1
bin750_A32_Soil_2_scaffold_916_c1_20
bin750_A32_Soil_1_scaffold_1136_c1_6
uncultured_virus_CAH6420276.1
Satyrvirus_sp._AYV84990.1
Cotonvirus_japonicus_YP_010841719.1
IMGVR_UViG_3300020057_000404_3300020057_Ga0163151_10000006285
IMGVR_UViG_3300015163_000024_3300015163_Ga0167665_10009625
uncultured_virus_CAI2358523.1
Acanthamoeba_polyphaga_mimivirus_AKI79207.1
Acanthamoeba_polyphaga_mimivirus_gi_311977822_ref_YP_003986942.1
Acanthamoeba_polyphaga_mimivirus_YP_010777350.1
Faunusvirus_sp._AYV79293.1
Yasminevirus_sp._GU–2018_VBB18108.1
Dasosvirus_sp._AYV77429.1
Bodo_saltans_virus_YP_010778369.1
Catovirus_CTV1_ARF09327.1
Terrestrivirus_sp._AYV75832.1
Harvfovirus_sp._AYV80832.1
Hyperionvirus_sp._AYV84219.1
Edafosvirus_sp._AYV78256.1
Fadolivirus_1_YP_010803319.1
Indivirus_ILV1_ARF09858.1
Klosneuvirus_KNV1_ARF11165.1
Mimivirus_LCMiAC01_QBK88881.1
uncultured_virus_CAH6421609.1
Homavirus_sp._AYV82013.1
Mimivirus_LCMiAC02_QBK89233.1
Mimivirus_LCMiAC02_QBK88971.1
Hokovirus_HKV1_ARF10841.1
Chlorella_virus_XW01_ULY68517.1
Megaviridae_environmental_sample_QFG74530.1
Cafeteria_roenbergensis_virus_BV–PW1_gi_310831328_ref_YP_003969971.1
Megaviridae_environmental_sample_QFG73779.1
Polychytrium_aggregatum_XP_052961513.1_AGB–1
Organic_Lake_phycodnavirus_2_gi_322510960_gb_ADX06273.1
Organic_Lake_phycodnavirus_1_gi_322510542_gb_ADX05856.1
Yellowstone_lake_mimivirus_YP_009174062.1
Megaviridae_environmental_sample_QFG75116.1
Phaeocystis_globosa_virus_12T_gi_357289601_gb_AET72914.1
Phaeocystis_globosa_virus_12T_gi_357289600_gb_AET72913.1
Chlamydomonas_sp._UWO_241_KAG1657904.1
Pyramimonas_orientalis_virus_QOI90457.1_HWQ62_00321
Mimiviridae_sp._ChoanoV1_QDY52214.15_11
Heliothis_virescens_ascovirus_3e_gi_134287264_ref_YP_001110960.1
Spodoptera_frugiperda_ascovirus_1a_gi_115298612_ref_YP_762465.1
Trichoplusia_ni_ascovirus_2c_gi_116326781_ref_YP_803318.1
Invertebrate_iridescent_virus_6_gi_15078788_ref_NP_149538.1
Wiseana_iridescent_virus_gi_339906066_ref_YP_004732863.1
Invertebrate_iridescent_virus_3_gi_109287966_ref_YP_654660.1
Singapore_grouper_iridovirus_gi_56692771_ref_YP_164229.1
Frog_virus_3_gi_49237312_ref_YP_031593.1
Lymphocystis_disease_virus_–_isolate_China_gi_51870067_ref_YP_073620.1
Infectious_spleen_and_kidney_necrosis_virus_gi_19881528_ref_NP_612345.1
Lausannevirus_gi_327409704_ref_YP_004347124.1
Marseillevirus_gi_284504185_ref_YP_003406900.1
Ostreococcus_lucimarinus_virus_OlV5_YP_007674806.1
Ostreococcus_virus_OsV5_gi_163955071_ref_YP_001648175.1
Ostreococcus_lucimarinus_virus_OlV1_gi_313844065_ref_YP_004061728.1
Ostreococcus_tauri_virus_RT–2011_AFC35001.1
Micromonas_sp._RCC1109_virus_MpV1_gi_313768295_ref_YP_004061975.1
Micromonas_pusilla_virus_PL1_AET43649.1
Micromonas_pusilla_virus_SP1_gi_357542059_gb_AET84819.1
Dishui_Lake_phycodnavirus_3_QIG59674.1
Dishui_lake_phycodnavirus_1_YP_009465797.1
Dishui_Lake_phycodnavirus_4_QIG59980.1
Bathycoccus_sp._RCC1105_virus_BpV2_ADQ91246.1
Bathycoccus_sp._RCC1105_virus_BpV1_gi_313768078_ref_YP_004061509.1
Dishui_Lake_phycodnavirus_2_QIG59506.1
Yellowstone_lake_phycodnavirus_3_YP_009174374.1AR677
Yellowstone_lake_phycodnavirus_1_YP_009174839.1
Yellowstone_lake_phycodnavirus_2_YP_009174479.1
Paramecium_bursaria_Chlorella_virus_CvsA1_AGE52551.1
Acanthocystis_turfacea_Chlorella_virus_1_gi_155371384_ref_YP_001426918.1
Paramecium_bursaria_Chlorella_virus_NYs1_gi_448935227_gb_AGE58778.1
Pandoravirus_quercus_YP_009482967.1
Pandoravirus_dulcis_gi_526119111_ref_YP_008319014.1
Pandoravirus_dulcis_YP_008319014.2
Pandoravirus_belohorizontensis_UMO78270.1
Pandoravirus_japonicus_BCU03580.1
Pandoravirus_salinus_gi_516306303_gb_AGO85882.1
IMGVR_UViG_2716885049_000001_2716885049_2718201226
IMGVR_UViG_3300048852_000066_3300048852_Ga0496061_000023_365882_366886
Pandoravirus_massiliensis_UDO47781.1
bin800_A32_Soil_4_scaffold_1418_c1_12
Pandoravirus_macleodensis_YP_009480906.1
Pandoravirus_kuranda_WBR14389.1
Pandoravirus_neocaledonia_YP_009481848.1
IMGVR_UViG_3300048852_000066_3300048852_Ga0496061_000023_367166_367579
IMGVR_UViG_2974576589_000001_2974576589_2974576810
Pandoravirus_celtis_QBZ81671.1
Pandoravirus_quercus_YP_009483760.1
Pandoravirus_inopinatum_YP_009119120.1
Pandoravirus_salinus_gi_516303807_gb_AGO83386.1
Pandoravirus_salinus_gi_516303793_gb_AGO83372.1
Pandoravirus_salinus_gi_516305958_gb_AGO85537.1
Pandoravirus_japonicus_BCU03004.1
Pandoravirus_dulcis_gi_526119975_ref_YP_008319878.1
Pandoravirus_kuranda_WBR14942.1
Pandoravirus_neocaledonia_YP_009482486.1
Pandoravirus_massiliensis_UDO47034.1
Pandoravirus_aubagnensis_UMO80288.1
Pandoravirus_macleodensis_YP_009481487.1
bin800_A32_Soil_4_scaffold_1418_c1_13
IMGVR_UViG_3300042923_001726_3300042923_Ga0456365_0000007_649114_649722
IMGVR_UViG_3300042922_001412_3300042922_Ga0456363_0000006_646884_647360
IMGVR_UViG_3300037519_000437_3300037519_Ga0335044_0000011_72822_73709
IMGVR_UViG_3300020495_000003_3300020495_Ga0208720_100000146
Pandoravirus_salinus_gi_516305953_gb_AGO85532.1
IMGVR_UViG_GVMAG–S–3300010368–40_000001_3300010368_Ga0129324_100065486
Emiliania_huxleyi_virus_86_gi_73852542_ref_YP_293826.1
IMGVR_UViG_3300031825_000002_3300031825_Ga0255338_100014484
Klebsormidium_nitens_GAQ91699.1KFL_008360070_
Feldmannia_species_virus_gi_197322436_ref_YP_002154709.1
Ectocarpus_siliculosus_virus_1_gi_13242498_ref_NP_077511.1
IMGVR_UViG_3300049783_001540_3300049783_Ga0491198_0000153_53702_54730
IMGVR_UViG_3300042923_001788_3300042923_Ga0456365_0000114_104147_105187
IMGVR_UViG_3300030339_000013_3300030339_Ga0311360_1000009145
Pacmanvirus_S19_QYB17551.1PV–S19_0187
Pacmanvirus_A23_YP_009361543.1
IMGVR_UViG_3300043411_001634_3300043411_Ga0451708_0000007_84457_85482
IMGVR_UViG_3300043411_001602_3300043411_Ga0451708_0000032_61971_62990
IMGVR_UViG_3300037466_002091_3300037466_Ga0395898_0000116_55605_56630
IMGVR_UViG_3300031521_000123_3300031521_Ga0311364_1000069717
bin795_A32_Soil_3_scaffold_2084_c1_4
bin795_A32_Soil_1_scaffold_3497_c1_6
IMGVR_UViG_GVMAG–M–3300013133–40_000001_3300013133_Ga0172362_100026528
Faustovirus_AMN83767.1D5b_00345
Faustovirus_QJX72934.1
Faustovirus_AIB51863.1PRJ
IMGVR_UViG_3300024299_000008_3300024299_Ga0233448_100015341
African_swine_fever_virus_YP_009702812.1
African_swine_fever_virus_gi_9628186_ref_NP_042772.1
Abalone_asfa–like_virus_BBO54017.1
Acanthamoeba_polyphaga_moumouvirus_gi_441432609_ref_YP_007354651.1
Megavirus_chiliensis_gi_350611647_gb_AEQ33091.1
Acanthamoeba_castellanii_mamavirus_gi_351737817_gb_AEQ60852.1
Acanthamoeba_polyphaga_mimivirus_gi_311978049_ref_YP_003987169.1
Myxoma_virus_gi_9633756_ref_NP_051834.1
Yaba–like_disease_virus_gi_12085104_ref_NP_073506.1
Vaccinia_virus_gi_66275952_ref_YP_233037.1
Squirrelpox_virus_gi_549059730_emb_CCD83304.1
Orf_virus_gi_41057171_ref_NP_957885.1
Molluscum_contagiosum_virus_subtype_1_gi_9629072_ref_NP_044091.1
Canarypox_virus_gi_40556209_ref_NP_955294.1
Nile_crocodilepox_virus_gi_115531818_ref_YP_784344.1
Melanoplus_sanguinipes_entomopoxvirus_gi_9631462_ref_NP_048242.1
Anomala_cuprea_entomopoxvirus_gi_578887621_dbj_BAO49495.1
Mythimna_separata_entomopoxvirus_gi_506497908_ref_YP_008003707.1
Amsacta_moorei_entomopoxvirus_gi_9964464_ref_NP_064932.1
0.3

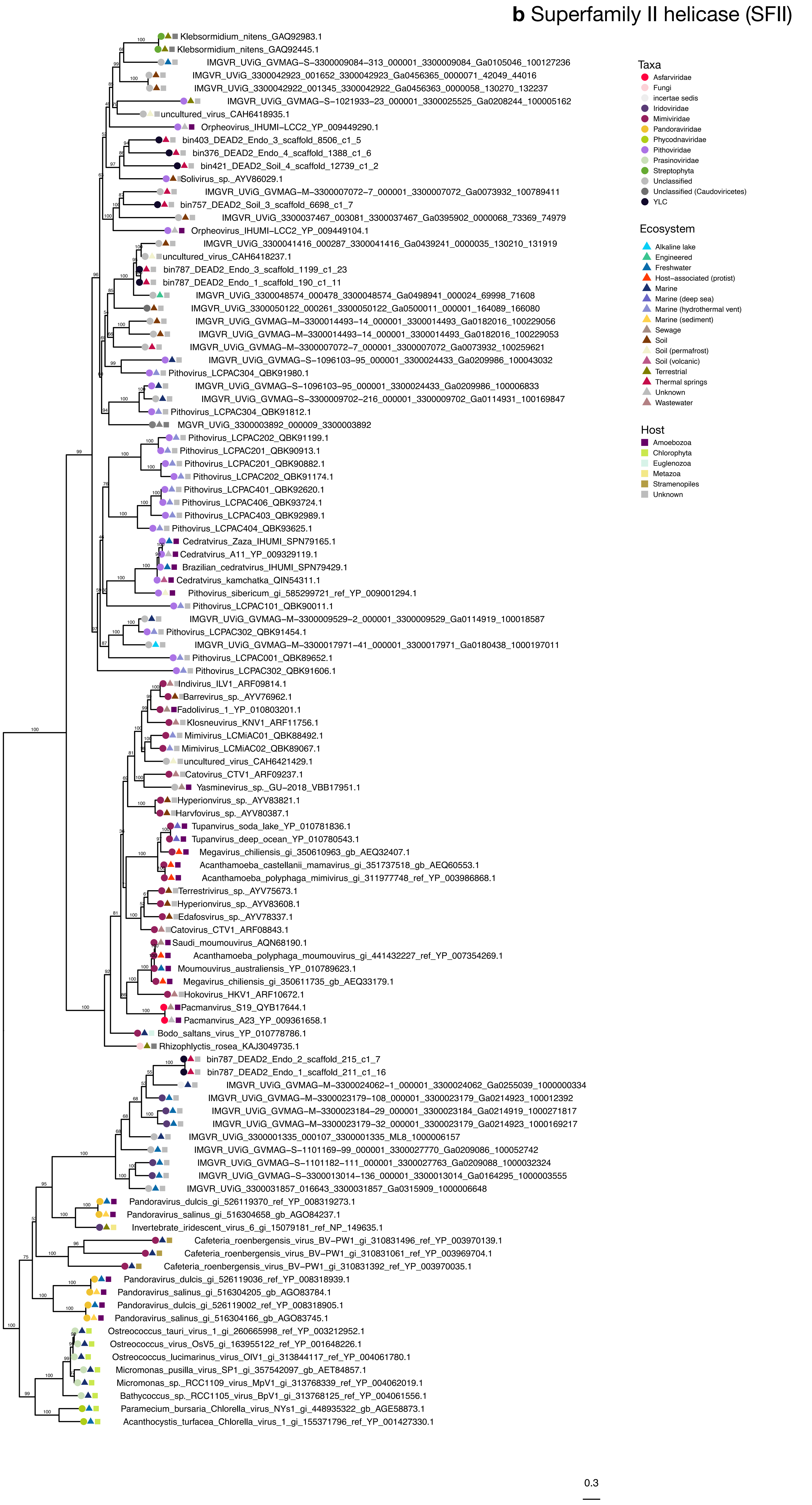
b Superfamily II helicase (SFII)
Klebsormidium_nitens_GAQ92983.1
Klebsormidium_nitens_GAQ92445.1
IMGVR_UViG_GVMAG–S–3300009084–313_000001_3300009084_Ga0105046_100127236
IMGVR_UViG_3300042923_001652_3300042923_Ga0456365_0000071_42049_44016
IMGVR_UViG_3300042922_001345_3300042922_Ga0456363_0000058_130270_132237
IMGVR_UViG_GVMAG–S–1021933–23_000001_3300025525_Ga0208244_100005162
uncultured_virus_CAH6418935.1
Orpheovirus_IHUMI–LCC2_YP_009449290.1
bin403_DEAD2_Endo_3_scaffold_8506_c1_5
bin376_DEAD2_Endo_4_scaffold_1388_c1_6
bin421_DEAD2_Soil_4_scaffold_12739_c1_2
Solivirus_sp._AYV86029.1
IMGVR_UViG_GVMAG–M–3300007072–7_000001_3300007072_Ga0073932_100789411
bin757_DEAD2_Soil_3_scaffold_6698_c1_7
IMGVR_UViG_3300037467_003081_3300037467_Ga0395902_0000068_73369_74979
Orpheovirus_IHUMI–LCC2_YP_009449104.1
IMGVR_UViG_3300041416_000287_3300041416_Ga0439241_0000035_130210_131919
uncultured_virus_CAH6418237.1
bin787_DEAD2_Endo_3_scaffold_1199_c1_23
bin787_DEAD2_Endo_1_scaffold_190_c1_11
IMGVR_UViG_3300048574_000478_3300048574_Ga0498941_000024_69998_71608
IMGVR_UViG_3300050122_000261_3300050122_Ga0500011_000001_164089_166080
IMGVR_UViG_GVMAG–M–3300014493–14_000001_3300014493_Ga0182016_100229056
IMGVR_UViG_GVMAG–M–3300014493–14_000001_3300014493_Ga0182016_100229053
IMGVR_UViG_GVMAG–M–3300007072–7_000001_3300007072_Ga0073932_100259621
IMGVR_UViG_GVMAG–S–1096103–95_000001_3300024433_Ga0209986_100043032
Pithovirus_LCPAC304_QBK91980.1
IMGVR_UViG_GVMAG–S–1096103–95_000001_3300024433_Ga0209986_100006833
IMGVR_UViG_GVMAG–S–3300009702–216_000001_3300009702_Ga0114931_100169847
Pithovirus_LCPAC304_QBK91812.1
MGVR_UViG_3300003892_000009_3300003892
Pithovirus_LCPAC202_QBK91199.1
Pithovirus_LCPAC201_QBK90913.1
Pithovirus_LCPAC201_QBK90882.1
Pithovirus_LCPAC202_QBK91174.1
Pithovirus_LCPAC401_QBK92620.1
Pithovirus_LCPAC406_QBK93724.1
Pithovirus_LCPAC403_QBK92989.1
Pithovirus_LCPAC404_QBK93625.1
Cedratvirus_Zaza_IHUMI_SPN79165.1
Cedratvirus_A11_YP_009329119.1
Brazilian_cedratvirus_IHUMI_SPN79429.1
Cedratvirus_kamchatka_QIN54311.1
Pithovirus_sibericum_gi_585299721_ref_YP_009001294.1
Pithovirus_LCPAC101_QBK90011.1
IMGVR_UViG_GVMAG–M–3300009529–2_000001_3300009529_Ga0114919_100018587
Pithovirus_LCPAC302_QBK91454.1
IMGVR_UViG_GVMAG–M–3300017971–41_000001_3300017971_Ga0180438_1000197011
Pithovirus_LCPAC001_QBK89652.1
Pithovirus_LCPAC302_QBK91606.1
Indivirus_ILV1_ARF09814.1
Barrevirus_sp._AYV76962.1
Fadolivirus_1_YP_010803201.1
Klosneuvirus_KNV1_ARF11756.1
Mimivirus_LCMiAC01_QBK88492.1
Mimivirus_LCMiAC02_QBK89067.1
uncultured_virus_CAH6421429.1
Catovirus_CTV1_ARF09237.1
Yasminevirus_sp._GU–2018_VBB17951.1
Hyperionvirus_sp._AYV83821.1
Harvfovirus_sp._AYV80387.1
Tupanvirus_soda_lake_YP_010781836.1
Tupanvirus_deep_ocean_YP_010780543.1
Megavirus_chiliensis_gi_350610963_gb_AEQ32407.1
Acanthamoeba_castellanii_mamavirus_gi_351737518_gb_AEQ60553.1
Acanthamoeba_polyphaga_mimivirus_gi_311977748_ref_YP_003986868.1
Terrestrivirus_sp._AYV75673.1
Hyperionvirus_sp._AYV83608.1
Edafosvirus_sp._AYV78337.1
Catovirus_CTV1_ARF08843.1
Saudi_moumouvirus_AQN68190.1
Acanthamoeba_polyphaga_moumouvirus_gi_441432227_ref_YP_007354269.1
Moumouvirus_australiensis_YP_010789623.1
Megavirus_chiliensis_gi_350611735_gb_AEQ33179.1
Hokovirus_HKV1_ARF10672.1
Pacmanvirus_S19_QYB17644.1
Pacmanvirus_A23_YP_009361658.1
Bodo_saltans_virus_YP_010778786.1
Rhizophlyctis_rosea_KAJ3049735.1
bin787_DEAD2_Endo_2_scaffold_215_c1_7
bin787_DEAD2_Endo_1_scaffold_211_c1_16
IMGVR_UViG_GVMAG–M–3300024062–1_000001_3300024062_Ga0255039_1000000334
IMGVR_UViG_GVMAG–M–3300023179–108_000001_3300023179_Ga0214923_100012392
IMGVR_UViG_GVMAG–M–3300023184–29_000001_3300023184_Ga0214919_1000271817
IMGVR_UViG_GVMAG–M–3300023179–32_000001_3300023179_Ga0214923_1000169217
IMGVR_UViG_3300001335_000107_3300001335_ML8_1000006157
IMGVR_UViG_GVMAG–S–1101169–99_000001_3300027770_Ga0209086_100052742
IMGVR_UViG_GVMAG–S–1101182–111_000001_3300027763_Ga0209088_1000032324
IMGVR_UViG_GVMAG–S–3300013014–136_000001_3300013014_Ga0164295_1000003555
IMGVR_UViG_3300031857_016643_3300031857_Ga0315909_1000006648
Pandoravirus_dulcis_gi_526119370_ref_YP_008319273.1
Pandoravirus_salinus_gi_516304658_gb_AGO84237.1
Invertebrate_iridescent_virus_6_gi_15079181_ref_NP_149635.1
Cafeteria_roenbergensis_virus_BV–PW1_gi_310831496_ref_YP_003970139.1
Cafeteria_roenbergensis_virus_BV–PW1_gi_310831061_ref_YP_003969704.1
Cafeteria_roenbergensis_virus_BV–PW1_gi_310831392_ref_YP_003970035.1
Pandoravirus_dulcis_gi_526119036_ref_YP_008318939.1
Pandoravirus_salinus_gi_516304205_gb_AGO83784.1
Pandoravirus_dulcis_gi_526119002_ref_YP_008318905.1
Pandoravirus_salinus_gi_516304166_gb_AGO83745.1
Ostreococcus_tauri_virus_1_gi_260665998_ref_YP_003212952.1
Ostreococcus_virus_OsV5_gi_163955122_ref_YP_001648226.1
Ostreococcus_lucimarinus_virus_OlV1_gi_313844117_ref_YP_004061780.1
Micromonas_pusilla_virus_SP1_gi_357542097_gb_AET84857.1
Micromonas_sp._RCC1109_virus_MpV1_gi_313768339_ref_YP_004062019.1
Bathycoccus_sp._RCC1105_virus_BpV1_gi_313768125_ref_YP_004061556.1
Paramecium_bursaria_Chlorella_virus_NYs1_gi_448935322_gb_AGE58873.1
Acanthocystis_turfacea_Chlorella_virus_1_gi_155371796_ref_YP_001427330.1
Taxa
Asfarviridae
Fungi
incertae sedis
Iridoviridae
Mimiviridae
Pandoraviridae
Phycodnaviridae
Pithoviridae
Prasinoviridae
Streptophyta
Unclassified
Unclassified (Caudoviricetes)
YLC
Ecosystem
Alkaline lake
Engineered
Freshwater
Host–associated (protist)
Marine
Marine (deep sea)
Marine (hydrothermal vent)
Marine (sediment)
Sewage
Soil
Soil (permafrost)
Soil (volcanic)
Terrestrial
Thermal springs
Unknown
Wastewater
Host
Amoebozoa
Chlorophyta
Euglenozoa
Metazoa
Stramenopiles
Unknown
0.3

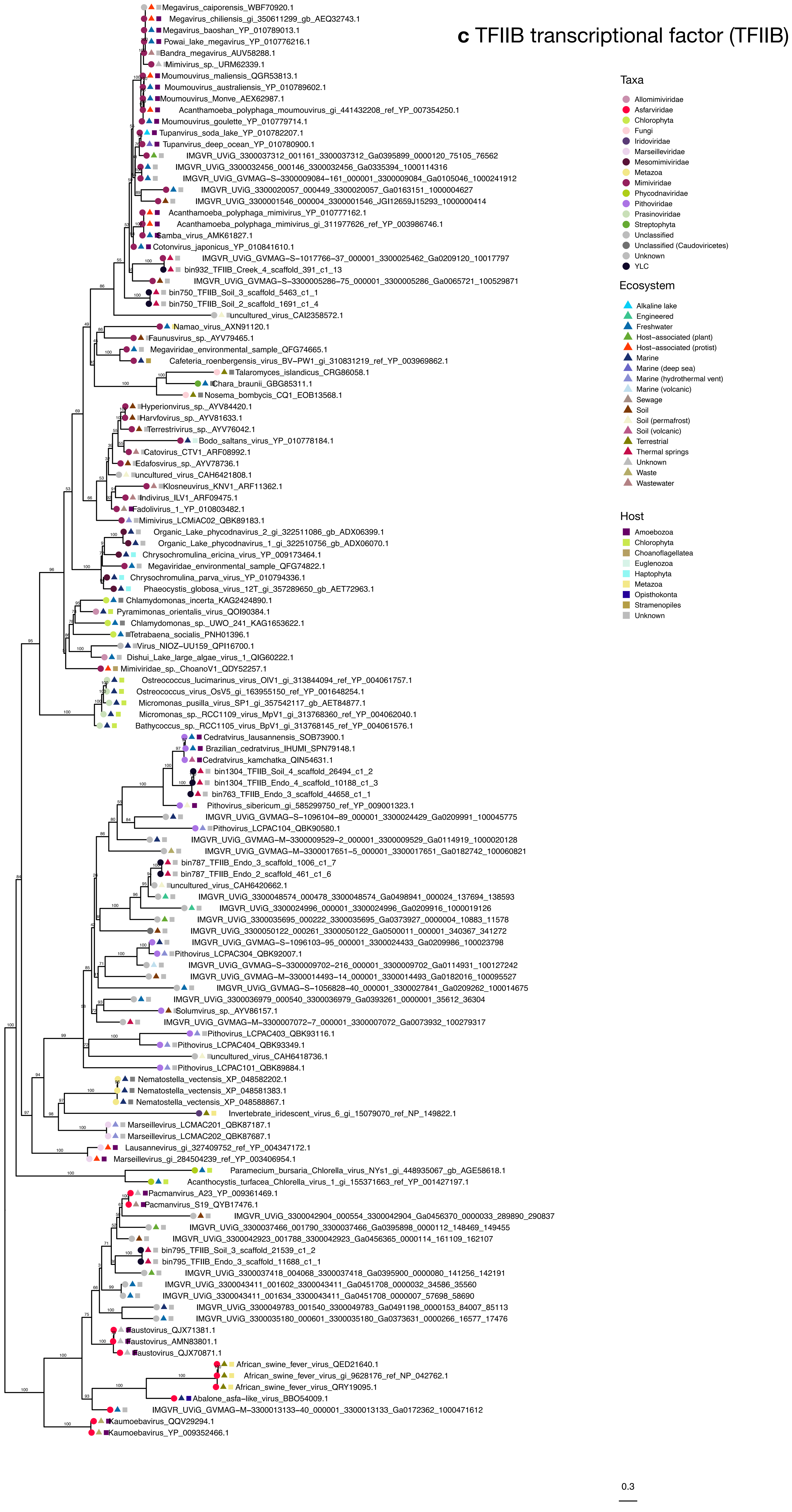
c TFIIB transcriptional factor (TFIIB)
Megavirus_caiporensis_WBF70920.1
Megavirus_chiliensis_gi_350611299_gb_AEQ32743.1
Megavirus_baoshan_YP_010789013.1
Powai_lake_megavirus_YP_010776216.1
Bandra_megavirus_AUV58288.1
Mimivirus_sp._URM62339.1
Moumouvirus_maliensis_QGR53813.1
Moumouvirus_australiensis_YP_010789602.1
Moumouvirus_Monve_AEX62987.1
Acanthamoeba_polyphaga_moumouvirus_gi_441432208_ref_YP_007354250.1
Moumouvirus_goulette_YP_010779714.1
Tupanvirus_soda_lake_YP_010782207.1
Tupanvirus_deep_ocean_YP_010780900.1
IMGVR_UViG_3300037312_001161_3300037312_Ga0395899_0000120_75105_76562
IMGVR_UViG_3300032456_000146_3300032456_Ga0335394_1000114316
IMGVR_UViG_GVMAG−S−3300009084−161_000001_3300009084_Ga0105046_1000241912
IMGVR_UViG_3300020057_000449_3300020057_Ga0163151_1000004627
IMGVR_UViG_3300001546_000004_3300001546_JGI12659J15293_1000000414
Acanthamoeba_polyphaga_mimivirus_YP_010777162.1
Acanthamoeba_polyphaga_mimivirus_gi_311977626_ref_YP_003986746.1
Samba_virus_AMK61827.1
Cotonvirus_japonicus_YP_010841610.1
IMGVR_UViG_GVMAG−S−1017766−37_000001_3300025462_Ga0209120_10017797
bin932_TFIIB_Creek_4_scaffold_391_c1_13
IMGVR_UViG_GVMAG−S−3300005286−75_000001_3300005286_Ga0065721_100529871
bin750_TFIIB_Soil_3_scaffold_5463_c1_1
bin750_TFIIB_Soil_2_scaffold_1691_c1_4
uncultured_virus_CAI2358572.1
Namao_virus_AXN91120.1
Faunusvirus_sp._AYV79465.1
Megaviridae_environmental_sample_QFG74665.1
Cafeteria_roenbergensis_virus_BV−PW1_gi_310831219_ref_YP_003969862.1
Talaromyces_islandicus_CRG86058.1
Chara_braunii_GBG85311.1
Nosema_bombycis_CQ1_EOB13568.1
Hyperionvirus_sp._AYV84420.1
Harvfovirus_sp._AYV81633.1
Terrestrivirus_sp._AYV76042.1
Bodo_saltans_virus_YP_010778184.1
Catovirus_CTV1_ARF08992.1
Edafosvirus_sp._AYV78736.1
uncultured_virus_CAH6421808.1
Klosneuvirus_KNV1_ARF11362.1
Indivirus_ILV1_ARF09475.1
Fadolivirus_1_YP_010803482.1
Mimivirus_LCMiAC02_QBK89183.1
Organic_Lake_phycodnavirus_2_gi_322511086_gb_ADX06399.1
Organic_Lake_phycodnavirus_1_gi_322510756_gb_ADX06070.1
Chrysochromulina_ericina_virus_YP_009173464.1
Megaviridae_environmental_sample_QFG74822.1
Chrysochromulina_parva_virus_YP_010794336.1
Phaeocystis_globosa_virus_12T_gi_357289650_gb_AET72963.1
Chlamydomonas_incerta_KAG2424890.1
Pyramimonas_orientalis_virus_QOI90384.1
Chlamydomonas_sp._UWO_241_KAG1653622.1
Tetrabaena_socialis_PNH01396.1
Virus_NIOZ−UU159_QPI16700.1
Dishui_Lake_large_algae_virus_1_QIG60222.1
Mimiviridae_sp._ChoanoV1_QDY52257.1
Ostreococcus_lucimarinus_virus_OlV1_gi_313844094_ref_YP_004061757.1
Ostreococcus_virus_OsV5_gi_163955150_ref_YP_001648254.1
Micromonas_pusilla_virus_SP1_gi_357542117_gb_AET84877.1
Micromonas_sp._RCC1109_virus_MpV1_gi_313768360_ref_YP_004062040.1
Bathycoccus_sp._RCC1105_virus_BpV1_gi_313768145_ref_YP_004061576.1
Cedratvirus_lausannensis_SOB73900.1
Brazilian_cedratvirus_IHUMI_SPN79148.1
Cedratvirus_kamchatka_QIN54631.1
bin1304_TFIIB_Soil_4_scaffold_26494_c1_2
bin1304_TFIIB_Endo_4_scaffold_10188_c1_3
bin763_TFIIB_Endo_3_scaffold_44658_c1_1
Pithovirus_sibericum_gi_585299750_ref_YP_009001323.1
IMGVR_UViG_GVMAG−S−1096104−89_000001_3300024429_Ga0209991_100045775
Pithovirus_LCPAC104_QBK90580.1
IMGVR_UViG_GVMAG−M−3300009529−2_000001_3300009529_Ga0114919_1000020128
IMGVR_UViG_GVMAG−M−3300017651−5_000001_3300017651_Ga0182742_100060821
bin787_TFIIB_Endo_3_scaffold_1006_c1_7
bin787_TFIIB_Endo_2_scaffold_461_c1_6
uncultured_virus_CAH6420662.1
IMGVR_UViG_3300048574_000478_3300048574_Ga0498941_000024_137694_138593
IMGVR_UViG_3300024996_000001_3300024996_Ga0209916_1000019126
IMGVR_UViG_3300035695_000222_3300035695_Ga0373927_0000004_10883_11578
IMGVR_UViG_3300050122_000261_3300050122_Ga0500011_000001_340367_341272
IMGVR_UViG_GVMAG−S−1096103−95_000001_3300024433_Ga0209986_100023798
Pithovirus_LCPAC304_QBK92007.1
IMGVR_UViG_GVMAG−S−3300009702−216_000001_3300009702_Ga0114931_100127242
IMGVR_UViG_GVMAG−M−3300014493−14_000001_3300014493_Ga0182016_100095527
IMGVR_UViG_GVMAG−S−1056828−40_000001_3300027841_Ga0209262_100014675
IMGVR_UViG_3300036979_000540_3300036979_Ga0393261_0000001_35612_36304
Solumvirus_sp._AYV86157.1
IMGVR_UViG_GVMAG−M−3300007072−7_000001_3300007072_Ga0073932_100279317
Pithovirus_LCPAC403_QBK93116.1
Pithovirus_LCPAC404_QBK93349.1
uncultured_virus_CAH6418736.1
Pithovirus_LCPAC101_QBK89884.1
Nematostella_vectensis_XP_048582202.1
Nematostella_vectensis_XP_048581383.1
Nematostella_vectensis_XP_048588867.1
Invertebrate_iridescent_virus_6_gi_15079070_ref_NP_149822.1
Marseillevirus_LCMAC201_QBK87187.1
Marseillevirus_LCMAC202_QBK87687.1
Lausannevirus_gi_327409752_ref_YP_004347172.1
Marseillevirus_gi_284504239_ref_YP_003406954.1
Paramecium_bursaria_Chlorella_virus_NYs1_gi_448935067_gb_AGE58618.1
Acanthocystis_turfacea_Chlorella_virus_1_gi_155371663_ref_YP_001427197.1
Pacmanvirus_A23_YP_009361469.1
Pacmanvirus_S19_QYB17476.1
IMGVR_UViG_3300042904_000554_3300042904_Ga0456370_0000033_289890_290837
IMGVR_UViG_3300037466_001790_3300037466_Ga0395898_0000112_148469_149455
IMGVR_UViG_3300042923_001788_3300042923_Ga0456365_0000114_161109_162107
bin795_TFIIB_Soil_3_scaffold_21539_c1_2
bin795_TFIIB_Endo_3_scaffold_11688_c1_1
IMGVR_UViG_3300037418_004068_3300037418_Ga0395900_0000080_141256_142191
IMGVR_UViG_3300043411_001602_3300043411_Ga0451708_0000032_34586_35560
IMGVR_UViG_3300043411_001634_3300043411_Ga0451708_0000007_57698_58690
IMGVR_UViG_3300049783_001540_3300049783_Ga0491198_0000153_84007_85113
IMGVR_UViG_3300035180_000601_3300035180_Ga0373631_0000266_16577_17476
Faustovirus_QJX71381.1
Faustovirus_AMN83801.1
Faustovirus_QJX70871.1
African_swine_fever_virus_QED21640.1
African_swine_fever_virus_gi_9628176_ref_NP_042762.1
African_swine_fever_virus_QRY19095.1
Abalone_asfa−like_virus_BBO54009.1
IMGVR_UViG_GVMAG−M−3300013133−40_000001_3300013133_Ga0172362_1000471612
Kaumoebavirus_QQV29294.1
Kaumoebavirus_YP_009352466.1
Taxa
Allomimiviridae
Asfarviridae
Chlorophyta
Fungi
Iridoviridae
Marseilleviridae
Mesomimiviridae
Metazoa
Mimiviridae
Phycodnaviridae
Pithoviridae
Prasinoviridae
Streptophyta
Unclassified
Unclassified (Caudoviricetes)
Unknown
YLC
Ecosystem
Alkaline lake
Engineered
Freshwater
Host−associated (plant)
Host−associated (protist)
Marine
Marine (deep sea)
Marine (hydrothermal vent)
Marine (volcanic)
Sewage
Soil
Soil (permafrost)
Soil (volcanic)
Terrestrial
Thermal springs
Unknown
Waste
Wastewater
Host
Amoebozoa
Chlorophyta
Choanoflagellatea
Euglenozoa
Haptophyta
Metazoa
Opisthokonta
Stramenopiles
Unknown
0.3

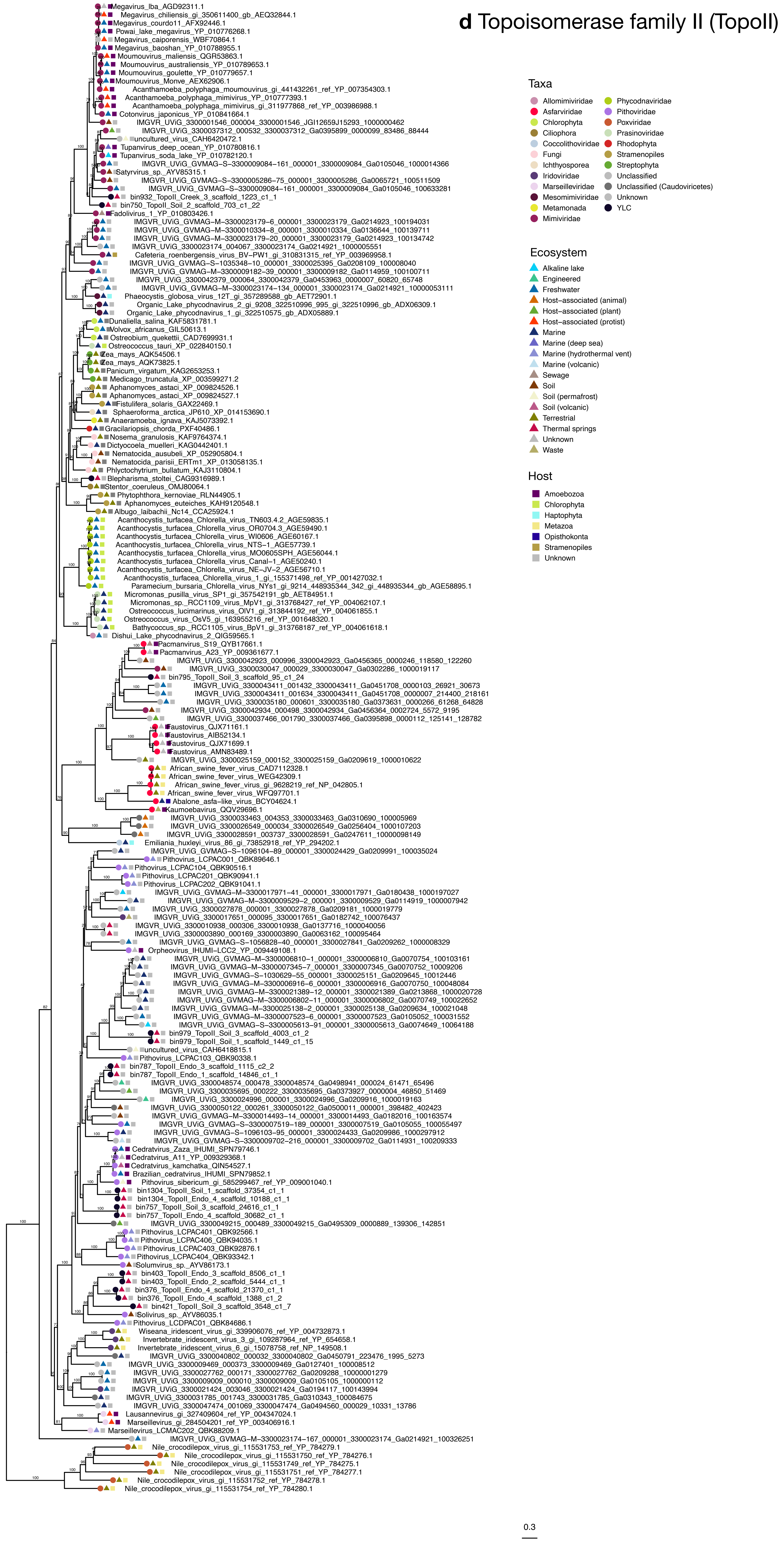
d Topoisomerase family II (TopoII)
Taxa
Allomimiviridae
Asfarviridae
Chlorophyta
Ciliophora
Coccolithoviridae
Fungi
Ichthyosporea
Iridoviridae
Marseilleviridae
Mesomimiviridae
Metamonada
Mimiviridae
Phycodnaviridae
Pithoviridae
Poxviridae
Prasinoviridae
Rhodophyta
Stramenopiles
Streptophyta
Unclassified
Unclassified (Caudoviricetes)
Unknown
YLC
Ecosystem
Alkaline lake
Engineered
Freshwater
Host–associated (animal)
Host–associated (plant)
Host–associated (protist)
Marine
Marine (deep sea)
Marine (hydrothermal vent)
Marine (volcanic)
Sewage
Soil
Soil (permafrost)
Soil (volcanic)
Terrestrial
Thermal springs
Unknown
Waste
Host
Amoebozoa
Chlorophyta
Haptophyta
Metazoa
Opisthokonta
Stramenopiles
Unknown
Megavirus_lba_AGD92311.1
Megavirus_chiliensis_gi_350611400_gb_AEQ32844.1
Megavirus_courdo11_AFX92446.1
Powai_lake_megavirus_YP_010776268.1
Megavirus_caiporensis_WBF70864.1
Megavirus_baoshan_YP_010788955.1
Moumouvirus_maliensis_QGR53863.1
Moumouvirus_australiensis_YP_010789653.1
Moumouvirus_goulette_YP_010779657.1
Moumouvirus_Monve_AEX62906.1
Acanthamoeba_polyphaga_moumouvirus_gi_441432261_ref_YP_007354303.1
Acanthamoeba_polyphaga_mimivirus_YP_010777393.1
Acanthamoeba_polyphaga_mimivirus_gi_311977868_ref_YP_003986988.1
Cotonvirus_japonicus_YP_010841664.1
IMGVR_UViG_3300001546_000004_3300001546_JGI12659J15293_1000000462
IMGVR_UViG_3300037312_000532_3300037312_Ga0395899_0000099_83486_88444
uncultured_virus_CAH6420472.1
Tupanvirus_deep_ocean_YP_010780816.1
Tupanvirus_soda_lake_YP_010782120.1
IMGVR_UViG_GVMAG–S–3300009084–161_000001_3300009084_Ga0105046_1000014366
Satyrvirus_sp._AYV85315.1
IMGVR_UViG_GVMAG–S–3300005286–75_000001_3300005286_Ga0065721_100511509
IMGVR_UViG_GVMAG–S–3300009084–161_000001_3300009084_Ga0105046_100633281
bin932_TopoII_Creek_3_scaffold_1223_c1_1
bin750_TopoII_Soil_2_scaffold_703_c1_22
Fadolivirus_1_YP_010803426.1
IMGVR_UViG_GVMAG–M–3300023179–6_000001_3300023179_Ga0214923_100194031
IMGVR_UViG_GVMAG–M–3300010334–8_000001_3300010334_Ga0136644_100139711
IMGVR_UViG_GVMAG–M–3300023179–20_000001_3300023179_Ga0214923_100134742
IMGVR_UViG_3300023174_004067_3300023174_Ga0214921_1000005551
Cafeteria_roenbergensis_virus_BV–PW1_gi_310831315_ref_YP_003969958.1
IMGVR_UViG_GVMAG–S–1035348–10_000001_3300025395_Ga0208109_100008040
IMGVR_UViG_GVMAG–M–3300009182–39_000001_3300009182_Ga0114959_100100711
IMGVR_UViG_3300042379_000064_3300042379_Ga0453963_0000007_60820_65748
IMGVR_UViG_GVMAG–M–3300023174–134_000001_3300023174_Ga0214921_10000053111
Phaeocystis_globosa_virus_12T_gi_357289588_gb_AET72901.1
Organic_Lake_phycodnavirus_2_gi_9208_322510996_995_gi_322510996_gb_ADX06309.1
Organic_Lake_phycodnavirus_1_gi_322510575_gb_ADX05889.1
Dunaliella_salina_KAF5831781.1
Volvox_africanus_GIL50613.1
Ostreobium_quekettii_CAD7699931.1
Ostreococcus_tauri_XP_022840150.1
Zea_mays_AQK54506.1
Zea_mays_AQK73825.1
Panicum_virgatum_KAG2653253.1
Medicago_truncatula_XP_003599271.2
Aphanomyces_astaci_XP_009824526.1
Aphanomyces_astaci_XP_009824527.1
Fistulifera_solaris_GAX22469.1
Sphaeroforma_arctica_JP610_XP_014153690.1
Anaeramoeba_ignava_KAJ5073392.1
Gracilariopsis_chorda_PXF40486.1
Nosema_granulosis_KAF9764374.1
Dictyocoela_muelleri_KAG0442401.1
Nematocida_ausubeli_XP_052905804.1
Nematocida_parisii_ERTm1_XP_013058135.1
Phlyctochytrium_bullatum_KAJ3110804.1
Blepharisma_stoltei_CAG9316989.1
Stentor_coeruleus_OMJ80064.1
Phytophthora_kernoviae_RLN44905.1
Aphanomyces_euteiches_KAH9120548.1
Albugo_laibachii_Nc14_CCA25924.1
Acanthocystis_turfacea_Chlorella_virus_TN603.4.2_AGE59835.1
Acanthocystis_turfacea_Chlorella_virus_OR0704.3_AGE59490.1
Acanthocystis_turfacea_Chlorella_virus_WI0606_AGE60167.1
Acanthocystis_turfacea_Chlorella_virus_NTS–1_AGE57739.1
Acanthocystis_turfacea_Chlorella_virus_MO0605SPH_AGE56044.1
Acanthocystis_turfacea_Chlorella_virus_Canal–1_AGE50240.1
Acanthocystis_turfacea_Chlorella_virus_NE–JV–2_AGE56710.1
Acanthocystis_turfacea_Chlorella_virus_1_gi_155371498_ref_YP_001427032.1
Paramecium_bursaria_Chlorella_virus_NYs1_gi_9214_448935344_342_gi_448935344_gb_AGE58895.1
Micromonas_pusilla_virus_SP1_gi_357542191_gb_AET84951.1
Micromonas_sp._RCC1109_virus_MpV1_gi_313768427_ref_YP_004062107.1
Ostreococcus_lucimarinus_virus_OlV1_gi_313844192_ref_YP_004061855.1
Ostreococcus_virus_OsV5_gi_163955216_ref_YP_001648320.1
Bathycoccus_sp._RCC1105_virus_BpV1_gi_313768187_ref_YP_004061618.1
Dishui_Lake_phycodnavirus_2_QIG59565.1
Pacmanvirus_S19_QYB17661.1
Pacmanvirus_A23_YP_009361677.1
IMGVR_UViG_3300042923_000996_3300042923_Ga0456365_0000246_118580_122260
IMGVR_UViG_3300030047_000029_3300030047_Ga0302286_1000019117
bin795_TopoII_Soil_3_scaffold_95_c1_24
IMGVR_UViG_3300043411_001432_3300043411_Ga0451708_0000103_26921_30673
IMGVR_UViG_3300043411_001634_3300043411_Ga0451708_0000007_214400_218161
IMGVR_UViG_3300035180_000601_3300035180_Ga0373631_0000266_61268_64828
IMGVR_UViG_3300042934_000498_3300042934_Ga0456364_0002724_5572_9195
IMGVR_UViG_3300037466_001790_3300037466_Ga0395898_0000112_125141_128782
Faustovirus_QJX71161.1
Faustovirus_AIB52134.1
Faustovirus_QJX71699.1
Faustovirus_AMN83489.1
IMGVR_UViG_3300025159_000152_3300025159_Ga0209619_1000010622
African_swine_fever_virus_CAD7112328.1
African_swine_fever_virus_WEG42309.1
African_swine_fever_virus_gi_9628219_ref_NP_042805.1
African_swine_fever_virus_WFQ97701.1
Abalone_asfa–like_virus_BCY04624.1
Kaumoebavirus_QQV29696.1
IMGVR_UViG_3300033463_004353_3300033463_Ga0310690_100005969
IMGVR_UViG_3300026549_000034_3300026549_Ga0256404_1000107203
IMGVR_UViG_3300028591_003737_3300028591_Ga0247611_10000098149
Emiliania_huxleyi_virus_86_gi_73852918_ref_YP_294202.1
IMGVR_UViG_GVMAG–S–1096104–89_000001_3300024429_Ga0209991_100035024
Pithovirus_LCPAC001_QBK89646.1
Pithovirus_LCPAC104_QBK90516.1
Pithovirus_LCPAC201_QBK90941.1
Pithovirus_LCPAC202_QBK91041.1
IMGVR_UViG_GVMAG–M–3300017971–41_000001_3300017971_Ga0180438_1000197027
IMGVR_UViG_GVMAG–M–3300009529–2_000001_3300009529_Ga0114919_1000007942
IMGVR_UViG_3300027878_000001_3300027878_Ga0209181_1000019779
IMGVR_UViG_3300017651_000095_3300017651_Ga0182742_100076437
IMGVR_UViG_3300010938_000306_3300010938_Ga0137716_1000040056
IMGVR_UViG_3300003890_000169_3300003890_Ga0063162_100095464
IMGVR_UViG_GVMAG–S–1056828–40_000001_3300027841_Ga0209262_1000008329
Orpheovirus_IHUMI–LCC2_YP_009449108.1
IMGVR_UViG_GVMAG–M–3300006810–1_000001_3300006810_Ga0070754_100103161
IMGVR_UViG_GVMAG–M–3300007345–7_000001_3300007345_Ga0070752_10009206
IMGVR_UViG_GVMAG–S–1030629–55_000001_3300025151_Ga0209645_10012446
IMGVR_UViG_GVMAG–M–3300006916–6_000001_3300006916_Ga0070750_100048084
IMGVR_UViG_GVMAG–M–3300021389–12_000001_3300021389_Ga0213868_1000020728
IMGVR_UViG_GVMAG–M–3300006802–11_000001_3300006802_Ga0070749_100022652
IMGVR_UViG_GVMAG–M–3300025138–2_000001_3300025138_Ga0209634_100021048
IMGVR_UViG_GVMAG–M–3300007523–6_000001_3300007523_Ga0105052_100031552
IMGVR_UViG_GVMAG–S–3300005613–91_000001_3300005613_Ga0074649_10064188
bin979_TopoII_Soil_3_scaffold_4003_c1_2
bin979_TopoII_Soil_1_scaffold_1449_c1_15
uncultured_virus_CAH6418815.1
Pithovirus_LCPAC103_QBK90338.1
bin787_TopoII_Endo_3_scaffold_1115_c2_2
bin787_TopoII_Endo_1_scaffold_14846_c1_1
IMGVR_UViG_3300048574_000478_3300048574_Ga0498941_000024_61471_65496
IMGVR_UViG_3300035695_000222_3300035695_Ga0373927_0000004_46850_51469
IMGVR_UViG_3300024996_000001_3300024996_Ga0209916_1000019163
IMGVR_UViG_3300050122_000261_3300050122_Ga0500011_000001_398482_402423
IMGVR_UViG_GVMAG–M–3300014493–14_000001_3300014493_Ga0182016_100163574
IMGVR_UViG_GVMAG–S–3300007519–189_000001_3300007519_Ga0105055_100055497
IMGVR_UViG_GVMAG–S–1096103–95_000001_3300024433_Ga0209986_1000297912
IMGVR_UViG_GVMAG–S–3300009702–216_000001_3300009702_Ga0114931_100209333
Cedratvirus_Zaza_IHUMI_SPN79746.1
Cedratvirus_A11_YP_009329368.1
Cedratvirus_kamchatka_QIN54527.1
Brazilian_cedratvirus_IHUMI_SPN79852.1
Pithovirus_sibericum_gi_585299467_ref_YP_009001040.1
bin1304_TopoII_Soil_1_scaffold_37354_c1_1
bin1304_TopoII_Endo_4_scaffold_10188_c1_1
bin757_TopoII_Soil_3_scaffold_24616_c1_1
bin757_TopoII_Endo_4_scaffold_30682_c1_1
IMGVR_UViG_3300049215_000489_3300049215_Ga0495309_0000889_139306_142851
Pithovirus_LCPAC401_QBK92566.1
Pithovirus_LCPAC406_QBK94035.1
Pithovirus_LCPAC403_QBK92876.1
Pithovirus_LCPAC404_QBK93342.1
Solumvirus_sp._AYV86173.1
bin403_TopoII_Endo_3_scaffold_8506_c1_1
bin403_TopoII_Endo_2_scaffold_5444_c1_1
bin376_TopoII_Endo_4_scaffold_21370_c1_1
bin376_TopoII_Endo_4_scaffold_1388_c1_2
bin421_TopoII_Soil_3_scaffold_3548_c1_7
Solivirus_sp._AYV86035.1
Pithovirus_LCDPAC01_QBK84686.1
Wiseana_iridescent_virus_gi_339906076_ref_YP_004732873.1
Invertebrate_iridescent_virus_3_gi_109287964_ref_YP_654658.1
Invertebrate_iridescent_virus_6_gi_15078758_ref_NP_149508.1
IMGVR_UViG_3300040802_000032_3300040802_Ga0450791_223476_1995_5273
IMGVR_UViG_3300009469_000373_3300009469_Ga0127401_100008512
IMGVR_UViG_3300027762_000171_3300027762_Ga0209288_10000001279
IMGVR_UViG_3300009009_000010_3300009009_Ga0105105_1000000112
IMGVR_UViG_3300021424_003046_3300021424_Ga0194117_100143994
IMGVR_UViG_3300031785_001743_3300031785_Ga0310343_100084675
IMGVR_UViG_3300047474_001069_3300047474_Ga0494560_000029_10331_13786
Lausannevirus_gi_327409604_ref_YP_004347024.1
Marseillevirus_gi_284504201_ref_YP_003406916.1
Marseillevirus_LCMAC202_QBK88209.1
IMGVR_UViG_GVMAG–M–3300023174–167_000001_3300023174_Ga0214921_100326251
Nile_crocodilepox_virus_gi_115531753_ref_YP_784279.1
Nile_crocodilepox_virus_gi_115531750_ref_YP_784276.1
Nile_crocodilepox_virus_gi_115531749_ref_YP_784275.1
Nile_crocodilepox_virus_gi_115531751_ref_YP_784277.1
Nile_crocodilepox_virus_gi_115531752_ref_YP_784278.1
Nile_crocodilepox_virus_gi_115531754_ref_YP_784280.1
0.3

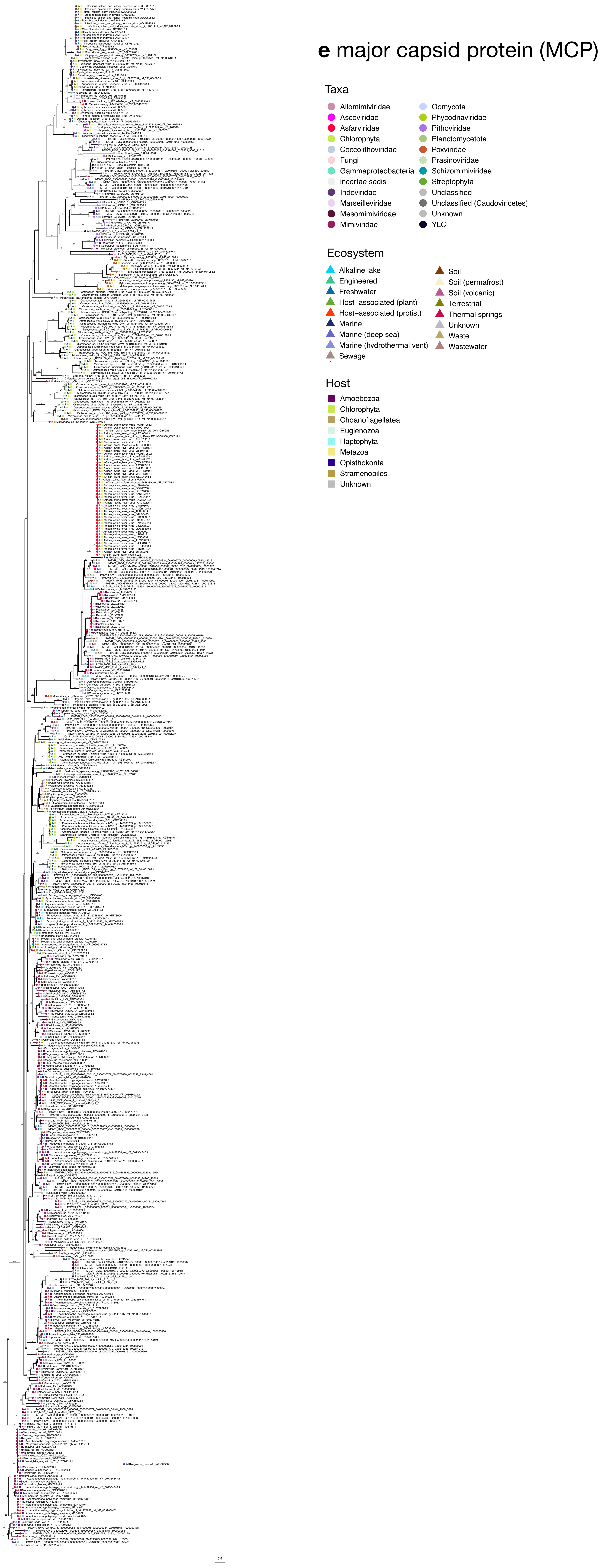
e major capsid protein (MCP)
Taxa
Allomimiviridae
Ascoviridae
Asfarviridae
Chlorophyta
Coccolithoviridae
Fungi
Gammaproteobacteria
incertae sedis
Iridoviridae
Marseilleviridae
Mesomimiviridae
Mimiviridae
Oomycota
Phycodnaviridae
Pithoviridae
Planctomycetota
Prasinoviridae
Poxviridae
Schizomimiviridae
Streptophyta
Unclassified
Unclassified (Caudoviricetes)
Unknown
YLC
Ecosystem
Alkaline lake
Engineered
Freshwater
Host-associated (plant)
Host-associated (protist)
Marine
Marine (deep sea)
Marine (hydrothermal vent)
Sewage
Soil
Soil (permafrost)
Soil (volcanic)
Terrestrial
Thermal springs
Unknown
Waste
Wastewater
Host
Amoebozoa
Chlorophyta
Choanoflagellatea
Euglenozoa
Haptophyta
Metazoa
Opisthokonta
Stramenopiles
Unknown
0.5

f virus late transcription factor 3 (VLTF3)

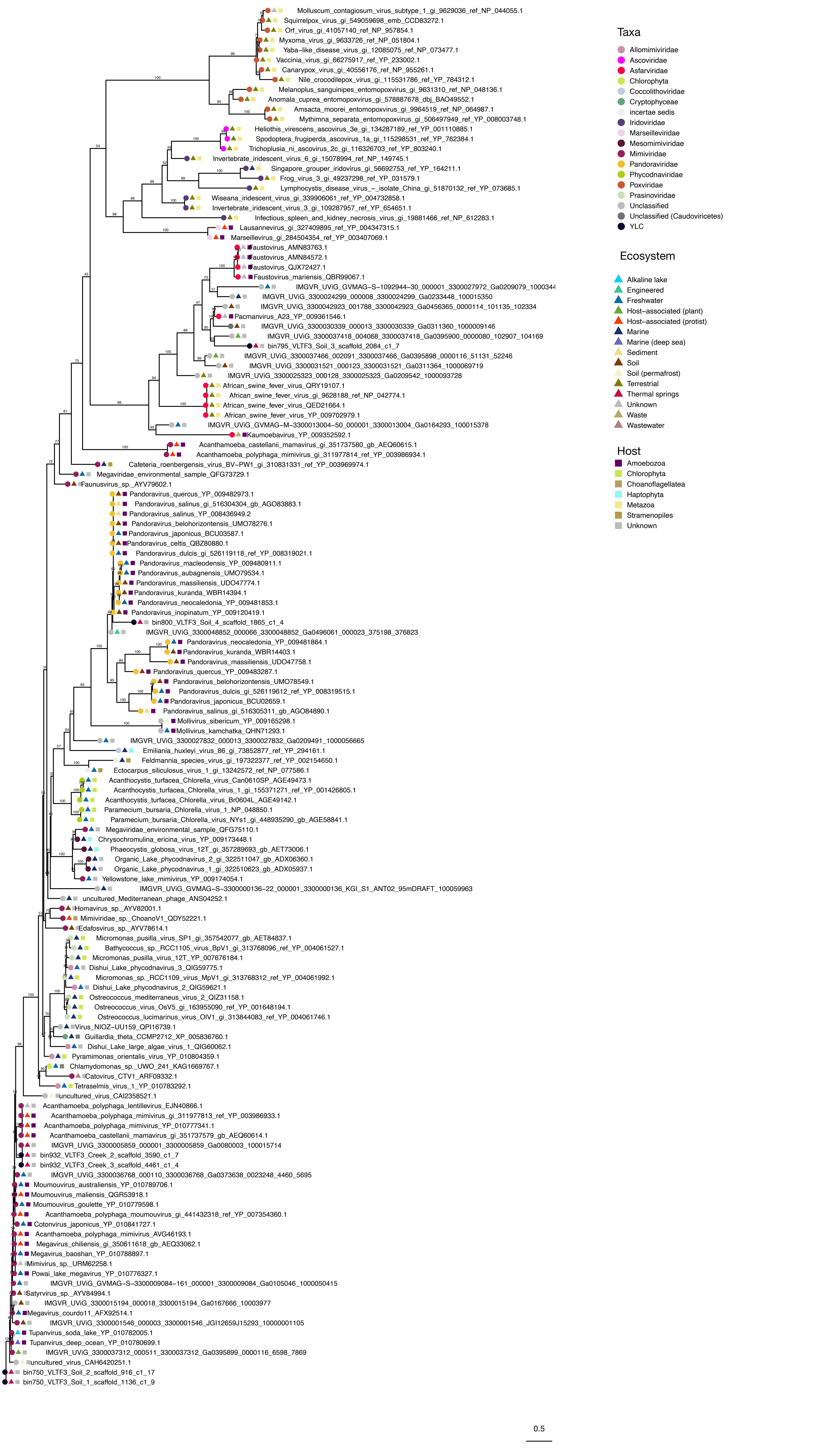

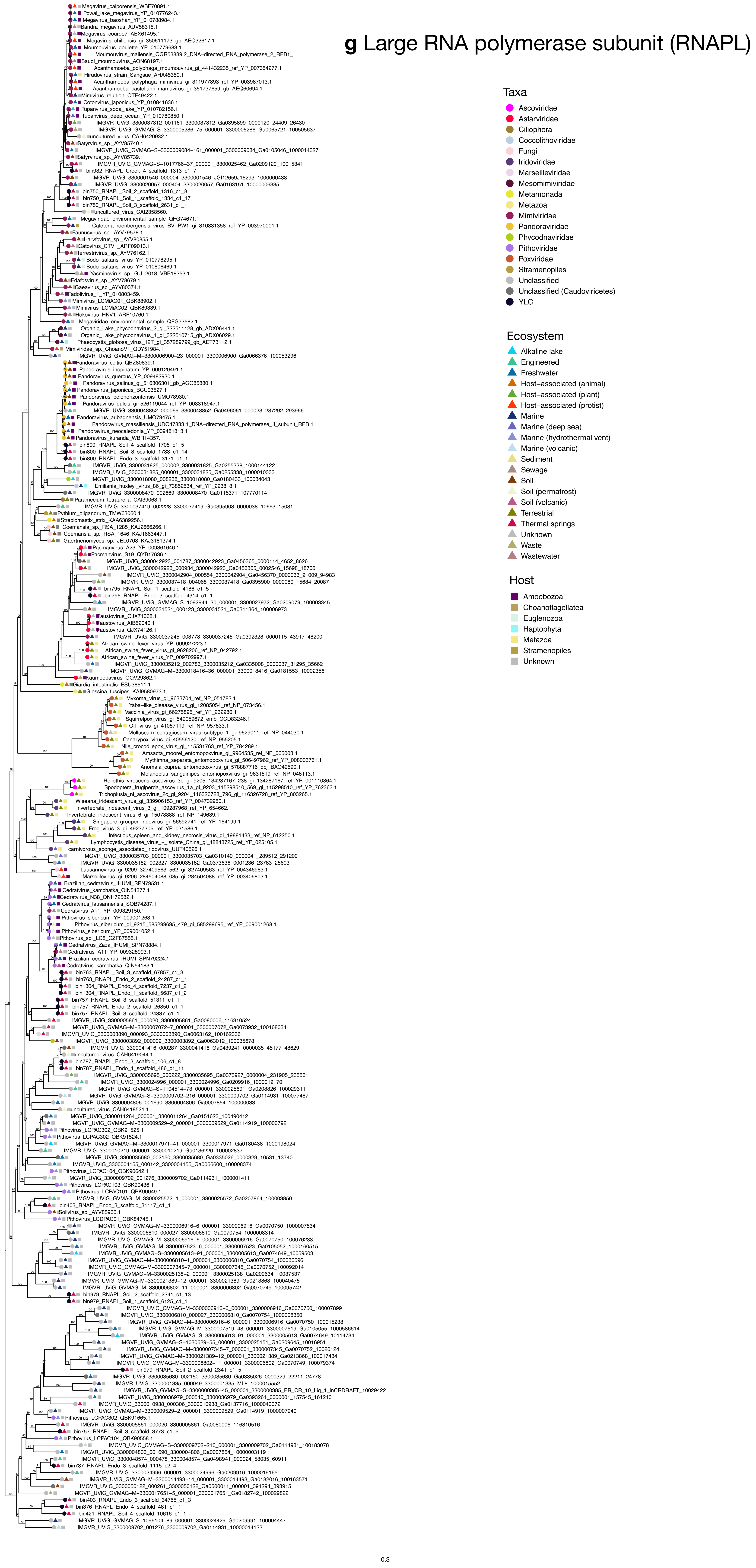
g Large RNA polymerase subunit (RNAPL)
Taxa
Ascoviridae
Asfarviridae
Ciliophora
Coccolithoviridae
Fungi
Iridoviridae
Marseilleviridae
Mesomimiviridae
Metamonada
Metazoa
Mimiviridae
Pandoraviridae
Phycodnaviridae
Pithoviridae
Poxviridae
Stramenopiles
Unclassified
Unclassified (Caudoviricetes)
YLC
Ecosystem
Alkaline lake
Engineered
Freshwater
Host−associated (animal)
Host−associated (plant)
Host−associated (protist)
Marine
Marine (deep sea)
Marine (hydrothermal vent)
Marine (volcanic)
Sediment
Sewage
Soil
Soil (permafrost)
Soil (volcanic)
Terrestrial
Thermal springs
Unknown
Waste
Wastewater
Host
Amoebozoa
Choanoflagellatea
Euglenozoa
Haptophyta
Metazoa
Stramenopiles
Unknown
0.3

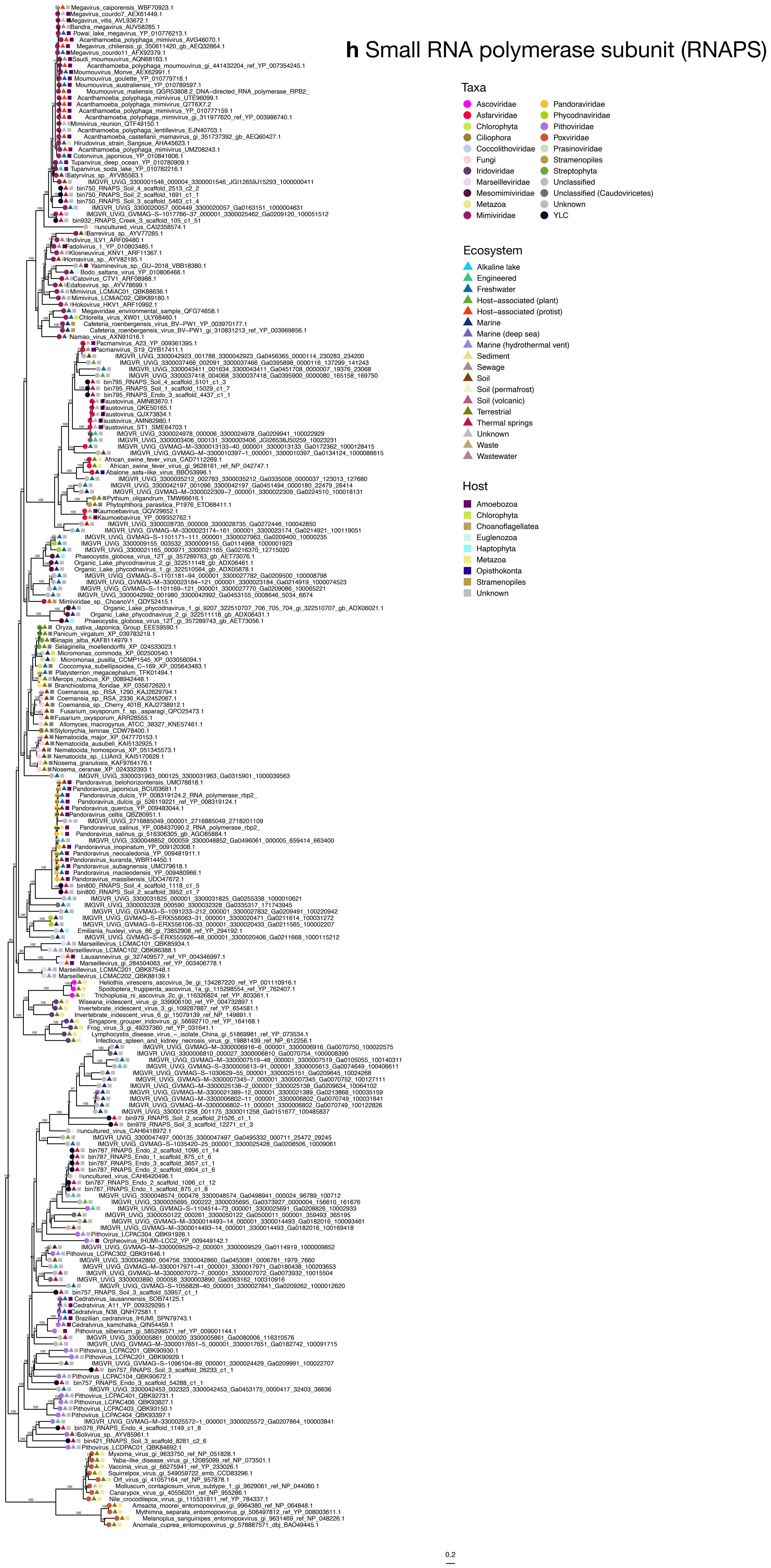
h Small RNA polymerase subunit (RNAPS)
Taxa
Ascoviridae
Asfarviridae
Chlorophyta
Ciliophora
Coccolithoviridae
Fungi
Iridoviridae
Marseilleviridae
Mesomimiviridae
Metazoa
Mimiviridae
Pandoraviridae
Phycodnaviridae
Pithoviridae
Poxviridae
Prasinoviridae
Stramenopiles
Streptophyta
Unclassified
Unclassified (Caudoviricetes)
Unknown
YLC
Ecosystem
Alkaline lake
Engineered
Freshwater
Host–associated (plant)
Host–associated (protist)
Marine
Marine (deep sea)
Marine (hydrothermal vent)
Sediment
Sewage
Soil
Soil (permafrost)
Soil (volcanic)
Terrestrial
Thermal springs
Unknown
Waste
Wastewater
Host
Amoebozoa
Chlorophyta
Choanoflagellatea
Euglenozoa
Haptophyta
Metazoa
Opisthokonta
Stramenopiles
Unknown
0.2

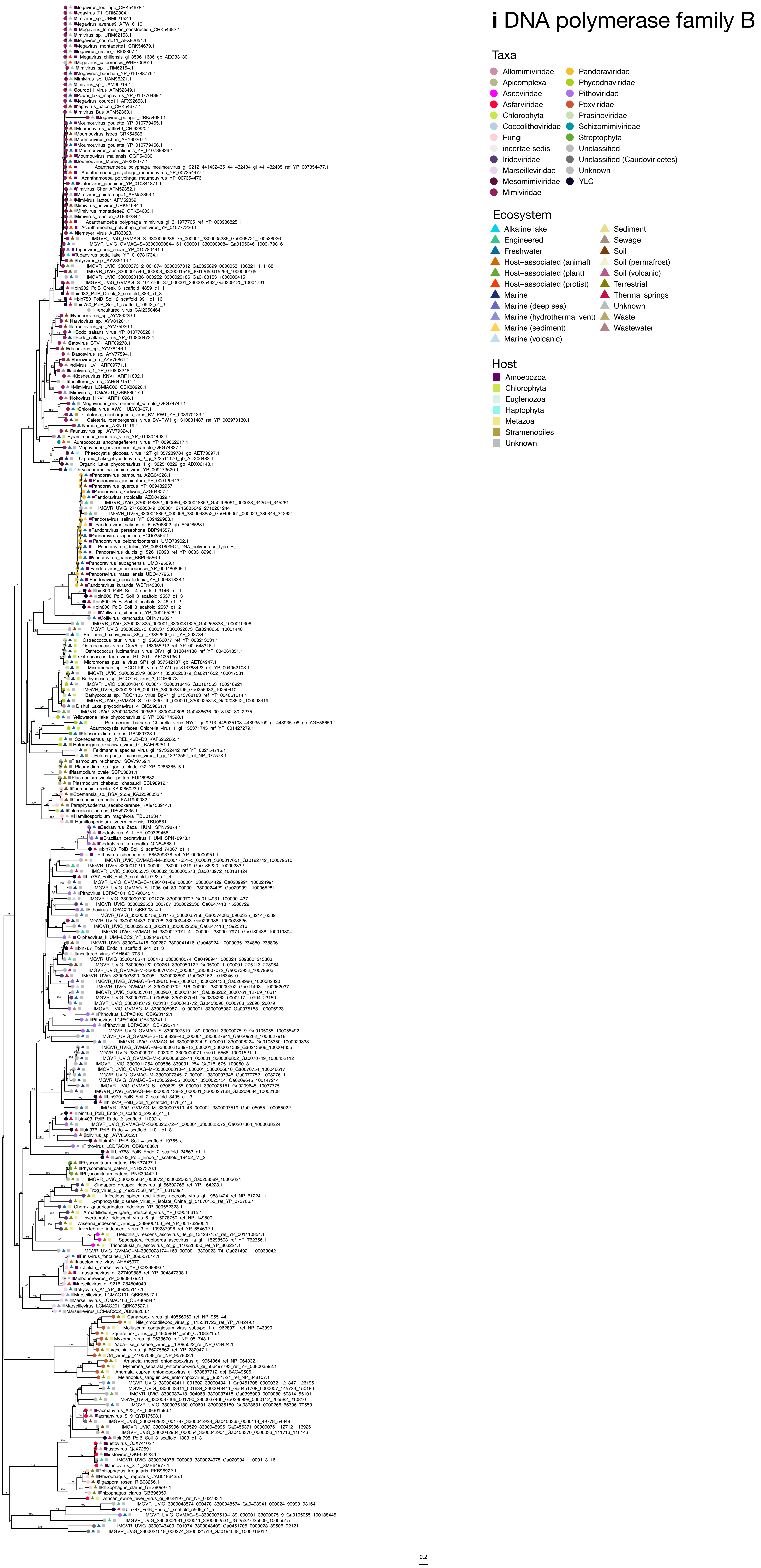

i DNA polymerase family B
Taxa
Allomimiviridae
Apicomplexa
Ascoviridae
Asfarviridae
Chlorophyta
Coccolithoviridae
Fungi
incertae sedis
Iridoviridae
Marseilleviridae
Mesomimiviridae
Mimiviridae
Pandoraviridae
Phycodnaviridae
Pithoviridae
Poxviridae
Prasinoviridae
Schizomimiviridae
Streptophyta
Unclassified
Unclassified (Caudoviricetes)
Unknown
YLC
Ecosystem
Alkaline lake
Engineered
Freshwater
Host-associated (animal)
Host-associated (plant)
Host-associated (protist)
Marine
Marine (deep sea)
Marine (hydrothermal vent)
Marine (sediment)
Marine (volcanic)
Sediment
Sewage
Soil
Soil (permafrost)
Soil (volcanic)
Terrestrial
Thermal springs
Unknown
Waste
Wastewater
Host
Amoebozoa
Chlorophyta
Euglenozoa
Haptophyta
Metazoa
Stramenopiles
Unknown
0.2
